# Genital malodor and olfactory reference syndrome: a resource for nonpsychiatrists

**DOI:** 10.1097/JW9.0000000000000228

**Published:** 2025-10-16

**Authors:** Alexa G. Ries, Padma Mohandas, Arucha L. Ekeowa-Anderson, Olushola L. Akinshemoyin Vaughn

**Affiliations:** a Department of Dermatology, Medical College of Wisconsin, Milwaukee, Wisconsin; b Department of Dermatology, Barts Health NHS Trust, London, UK

**Keywords:** Dermatology, genital malodor, olfactory reference syndrome, primary care, psychiatry

**What is known about this subject in regard to women and their families?**

- Genital malodor can present as a symptom of olfactory reference syndrome (ORS), which is a rare psychiatric disorder with significant morbidity and mortality. The severity of ORS appears to be higher in women, impacting not only the affected individuals but also their families. Sufferers perceive that they have genital malodor with no identifiable physical etiology.

**What is new from this article as messages for women and their families?**

- This article summarizes the clinical presentation of cases of ORS with genital malodor as a source of odor. The article provides a diagnostic and management approach for women presenting with genital malodor when ORS is suspected.

## Introduction

Genital malodor is a common symptom significantly impacting a woman’s quality of life. A third of women with genital malodor have no identifiable cause.^1^ If no objective findings of malodor are present and organic conditions, including COVID-19-related olfactory dysfunction, have been excluded, a psychiatric diagnosis of olfactory reference syndrome (ORS) may be considered. ORS is a primary psychiatric disorder in which the affected individual believes they emit an offensive odor when none is present, causing clinically significant distress.^2^ We aim to provide a practical approach for nonpsychiatrists to guide the diagnosis and management of ORS in the setting of genital malodor.

## Materials and methods

Peer-reviewed studies were identified through a PubMed search of ORS and genital malodor, followed by data extraction and analysis.

## Results

The largest studies of ORS include a systematic review, encompassing 84 cases, and a survey study of 253 patients with ORS.^2,3^ ORS is rare, and genital malodor is a source in 19 to 35% of reported cases (Table 1).^2,4^

**Table 1:** Characteristics of olfactory reference syndrome and genital malodor

N	Gender	Age of onset	Source of odor	Odor of concern	Associated symptoms	Author
253	• Female (32.8%) • Male (66.8%)	21.1	• Armpits (32.4%) • Feet (26.5%) • Breasts (22.1%) • Genitals (19.4%)	• Stool (26.5%) • Garbage (26.1%) • Ammonia (22.9%) • Metal (22.1%)	• Insight: Poor or delusional (18%) • Delusions of reference: (64%) • Olfactory hallucinations (59.3%)	Greenberg et al.^2^
84	• Female (38%) • Male (62%)	21	• Feet • Underarms • Groin • Sweat • Urine • Genital	• Sweat • Feces • Urine • Garbage • Dirty socks	• Insight: Delusional (57%) • Delusions of reference: (74%) • Olfactory hallucinations^a^ (22%)	Begum et al.^3^
20	• Female (60%) • Male (40%)	15.6	• Mouth (75%) • Armpits (60%) • Genitals (35%) • Anus (30%) • Feet (30%)	• Bad Breath (75%) • Sweat (65%) • Stool (30%) • Vaginal (10%)	• Insight: Delusional (85%) • Delusions of reference: (82.4%) • Olfactory hallucinations (85%)	Phillips et al.^4^

a Only 27 cases of 84 provided information about olfactory hallucinations. The percentage is of the 27 cases.

The mean age of onset of ORS ranges from 15 to 21 years old. Although ORS is more common in men, women have greater severity of symptoms and a greater number of sources of perceived odors, such as breath, armpits, and genitals.^2^ Presentations of ORS vary, from overvalued ideation, that is, difficulty acknowledging beliefs as untrue but can be reasoned with, to poor and absent insight with delusional beliefs. A majority of patients (74-97%) report lifetime ideas or delusions of reference, including misinterpretation of others’ verbal or nonverbal gestures.^2–4^ Excessive, repetitive behaviors can impair patients’ daily functioning, leading to social isolation and being housebound for days.^2^

Patients with ORS often do not seek help or treatment from a psychiatrist or psychologist but rather from other medical specialties (44%) and primary care physicians (11%).^5^ Of 84 cases of ORS, about half improved with treatment. Psychotherapy alone had the largest impact on improvement (78%), followed by antidepressants (58%), combined therapies (45%), and neuroleptics alone (33%).^3^

## Discussion

Patients with ORS and genital malodor often present to nonpsychiatric clinicians for management. This entity is important to recognize, especially in women who have an increased likelihood of severe disease.^2^ Although ORS is rare, morbidity and mortality are significant, with high rates of comorbid depression and suicidal ideation.^3,4^

**Fig. 1.**
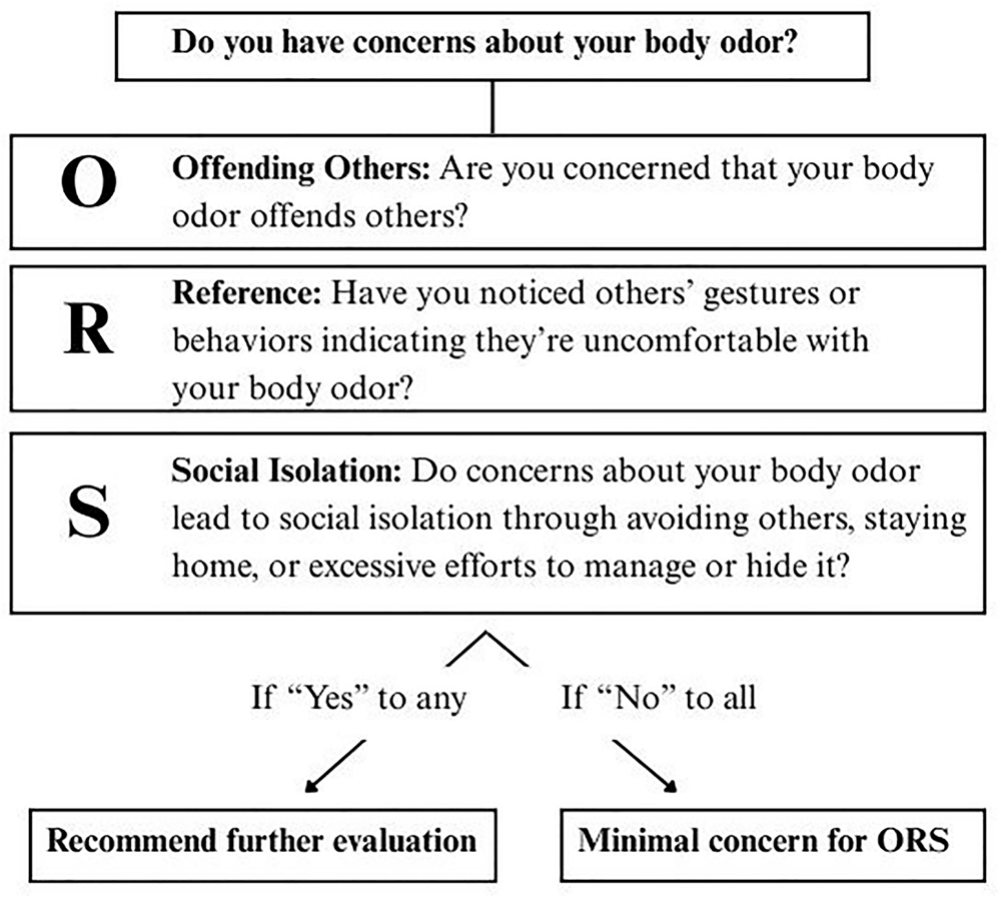
Screening tool for olfactory reference syndrome. Adapted with permission from Chernyak et al.^5^

An abbreviated screening tool may be utilized when suspecting ORS, especially in young patients with significant distress and decreased social functioning without objective findings of malodor (Fig. 1).^5^ Organic causes of genital malodor must be ruled out, and the workup should exclude common entities of vulvovaginitis, including bacterial vaginosis.^1^

Treatment guidelines for ORS are lacking, but studies suggest behavioral therapies and antidepressants may be the most effective.^2–5^ Selective serotonin reuptake inhibitors, including fluoxetine or sertraline, can be considered. These medications are nonaddictive and can help with obsessive thoughts and compulsive behaviors, with higher doses usually required for maintenance therapy. We advocate for an integrated multidisciplinary approach involving collaboration between mental health professionals and dermatology or gynecology professionals to improve patient outcomes.

## Conflict of interest

None.

## Funding

None.

## Study approval

N/A

## Author contribution

All authors participated in research design, writing of the paper, and data analysis.
